# Redox signaling induces laminin receptor ribosomal protein-SA expression to improve cell adhesion following radiofrequency glow discharge treatments

**DOI:** 10.1038/s41598-022-11766-9

**Published:** 2022-05-11

**Authors:** Sasikumar Ponnusamy, Hanan H. Ali, Felisha Dutt, Saeed Ur Rahman, Ahmad A. Salah, Mahek Pipalia, Robert E. Baier, Praveen R. Arany

**Affiliations:** 1 Department of Oral Biology, Surgery and Biomedical Engineering, University at Buffalo, 3435 Main Street, B36A Foster Hall, Buffalo, NY 14214 USA; 2 Department of Biomaterials, University at Buffalo, Buffalo, NY USA; 3 Oral Biology, Institute of Basic Medical Sciences, Khyber Medical University, Peshawar, 25000 Pakistan

**Keywords:** Animal biotechnology, Biomaterials, Tissue engineering, Materials science, Thermoelectric devices and materials, Translational research, Cell adhesion, Cell division

## Abstract

Current biomaterials effectively replace biological structures but are limited by infections and long-term material failures. This study examined the molecular mechanisms of radio frequency glow discharge treatments (RFGDT) in mediating the disinfection of biomaterial surfaces and concurrently promoting cell attachment and proliferation. Dental biomaterials were subjected to RFGDT, and viability of oral microbial species, namely *Streptococcus mutants* (SM), *Streptococcus gordonii* (SG), *Moraxella catarrhalis* (MC), and *Porphyromonas gingivalis* (PG), were assessed. Cell attachment and survival of a pre-odontoblast cell line, MDPC-23, was examined. Finally, mechanistic investigations into redox generation and biological signaling were investigated. Based on their compositions, dental biomaterials induced reactive oxygen species (ROS) following dose-dependent RFGDT. Reduced microbial viability was evident following RFGDT in the catalase-negative (SM and SG) species more prominently than catalase-positive (MC and PG) species. Cell adhesion assays noted improved MDPC-23 attachment and survival. Pretreatments with N-acetylcysteine (NAC) and catalase abrogated these responses. Immunoassays noted redox-induced downstream expression of a laminin receptor, Ribosomal Protein SA, following RFGDT. Thus, RFGDT-induced redox mediates antimicrobial and improves cell responses such as adhesion and proliferation. These observations together provide a mechanistic rationale for the clinical utility of RFGDT with dental biomaterials for regenerative clinical applications.

## Introduction

Biomaterials have enabled tissue engineering to evolve from an emerging science to its current pivotal role in spearheading clinical regenerative medicine^1–3^. These biomaterial systems have been readily adopted in various clinical fields for esthetics and functional replacements. There has been tremendous progress in biomaterial systems from passive, biocompatible carriers or inert replacements to current bioactive or *smart* systems capable of sense-and-respond capabilities^4,5^. Advances in these fields have encompassed newer compositions, optimized nanotopological features, biophysically-actuated and controlled-release biomaterial systems, among many others. These advancements are tied intimately with progress in development and stem cell biology, enabling a critical understanding of cell regulation during tissue healing and regeneration^6–8^. These include intrinsic regulation with key transcriptional factors, gene organization, signaling, metabolomics, and extrinsic regulation with membrane, nanoscale ligand engagement, and paracrine signaling. Moreover, among these extrinsic factors, the role of metagenomics from the pervasive microbiota is now better understood, further emphasizing the need for effective disinfection^9–13^.

The oral cavity presents several unique challenges with its soft and hard tissue biological constituents. These oral tissues represent an exclusive anatomical niche where hard tissue (teeth) is exposed through soft tissue (gingiva) via a delicate but rigorous interface. The importance of a healthy oral microbiome and the impact of dysbiosis in oral and systemic health has been well established^14–16^. These aspects present significant challenges for biomaterial replacements due to the constant biomechanical, microbiological assault, and immunological surveillance in the oral environment. The combination of physical stress, microbiological and host-derived enzymes, infections, and byproducts of degradation can all contribute synergistically to biomaterial failure^17,18^. Nonetheless, dentistry has very effectively utilized ceramics, metal, polymers, and their combinations as restorations, prostheses, or implants that are integral to current clinical care. Recent technologies such as digital impressions, high-resolution cone-beam computed tomography (CBCT), subtractive, and additive 3D printing have accelerated sophistication in device design and reduced production timelines enabling chair-side fabrication^19^. These advances with custom-fabricated biomaterial devices further emphasize the need to explore practical, in-office disinfection techniques.

An ideal approach must provide disinfection immediately prior to clinical use and not undermine physical or chemical material performance while optimally preparing the biomaterial interface to promote favorable host biological responses^20,21^. Various disinfection approaches have been employed from dry and wet heat, chemical disinfection, and biophysical means such as ultraviolet, ethylene dioxide gas, carbon dioxide, ultrasound, plasma, and microwaves^22,23^. Due to their device characteristics, several of these effective disinfection sources are restricted to commercial production and impractical for routine clinical use. Generation of plasma by glow discharge results from ionization of gaseous molecules termed the Townsend avalanche or discharge^24,25^. This process can be facilitated at atmospheric and low pressures resulting in a non-thermal process that minimally alters target surfaces. Several power sources have been utilized to generate glow-discharge plasma, such as direct current, electron cyclotron resonance, and radiofrequency. Among them, radiofrequency glow discharge treatment (RFGDT) is capable of generating a large volume of uniformly charged plasma that can flow through both conductive (metal) non-conductive (polymers) biomaterials^26,27^. RFGDT-generated plasma, in the presence of air or oxygen, removes organic contaminants by generating highly reactive oxygen species (ROS) that promote surface oxidation and hydroxylation^28^. RFGDT has also been noted to create high surface energy that increases hydrophilicity, facilitating cell spreading and attachment^27,29,30^. However, the precise mechanism mediating these improved cell adhesion following RFGDT has not been elucidated. This study investigated the effects of RFGDT on different dental biomaterial surfaces. The concurrent effects on disinfection of four common oral microbes and concurrent changes in cell adhesion and proliferation in an oral cell line, MDPC-23, were assessed. Finally, biochemical and molecular assays investigated the precise biological mechanism mediating the cellular responses.

## Results

### Redox generation at biomaterial interfaces following RFGDT

**Figure 1:**
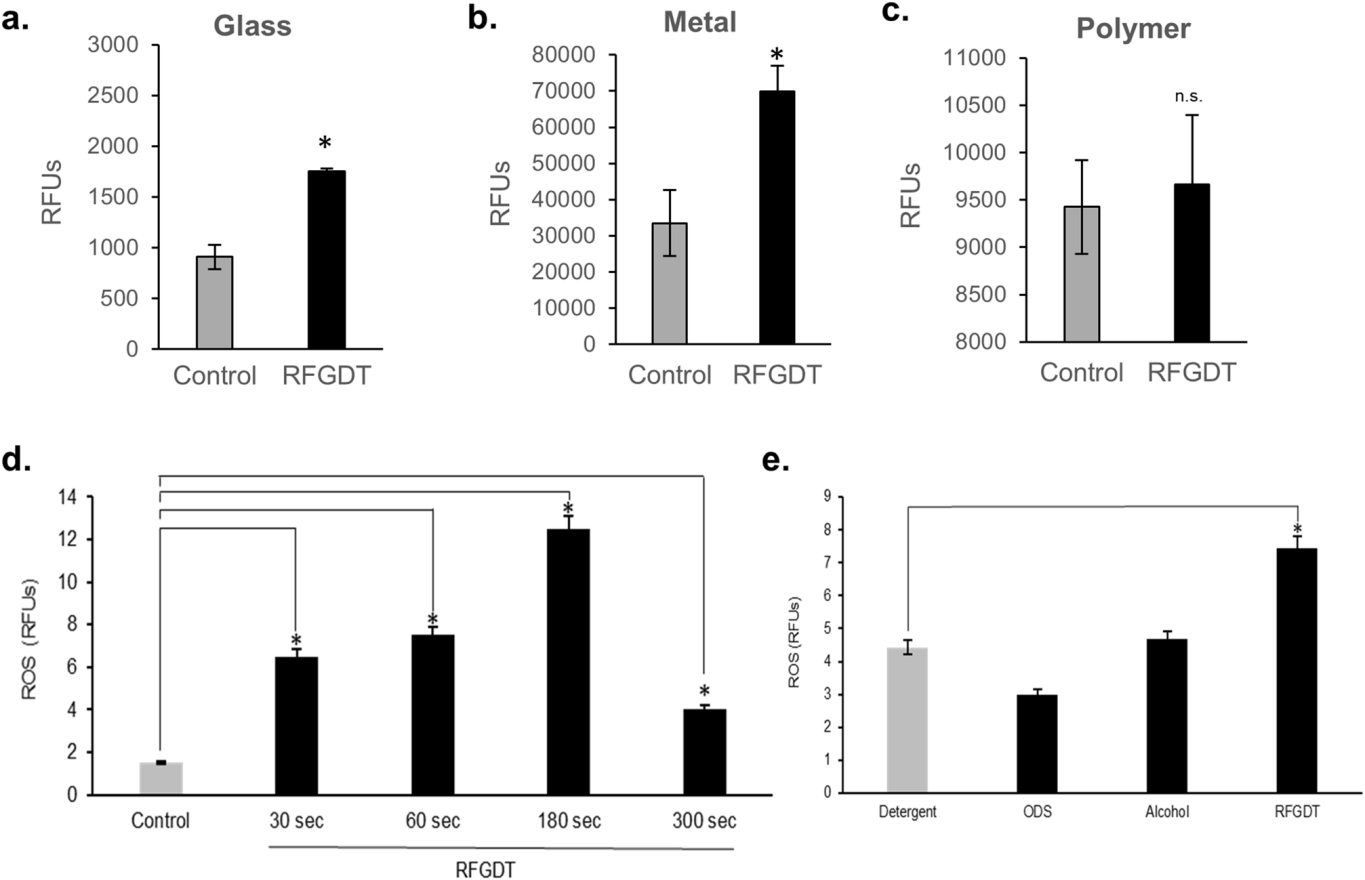
RFGDT generates redox from biomaterial surfaces. ROS generation was assessed with Luminol chemiluminescent assay immediately after RFGDT with various biomaterials namely (**a**) glass, (**b**) metal and (**c**) polymer; (**d**) ROS generation was also assessed following varying treatment times with RFGDT; (**e**) Glass surfaces pretreated with ODS, alcohol, or RFGDT were assessed with Luminol assay.

The plasma generated by RFGDT affects the surface chemistry of the biomaterials based on their composition and the presence of exogenous gases. As the dental pulp is exposed to various restorative biomaterials such as glass ionomer cement, polymers, and metals, we examined the generation of redox species following RFGDT with a chemiluminescence luminol assay^31^. RFGDT generated a significant increase in ROS from glass (Fig. 1a) and platinum surfaces (Fig. 1b), while minimal expression was noted on a polymeric (Polymethylmethacrylate, PMMA) surface (Fig. 1c). RFGDT induced a dose-dependent increase in redox generation with optimal noted at 180 s (Fig. 1d). As a prior study utilized various surface treatments such as detergent, alcohol, or silane, we noted that only RFGDT generates redox (Fig. 1e)^27^. These results show that while multiple agents can disinfect biomaterial interfaces, induction RFGDT-induced redox could persistently prevent microbial colonization while favorably modulating host cell interactions.

### Microbial colonization is inhibited by RFGDT-induced redox

**Figure 2:**
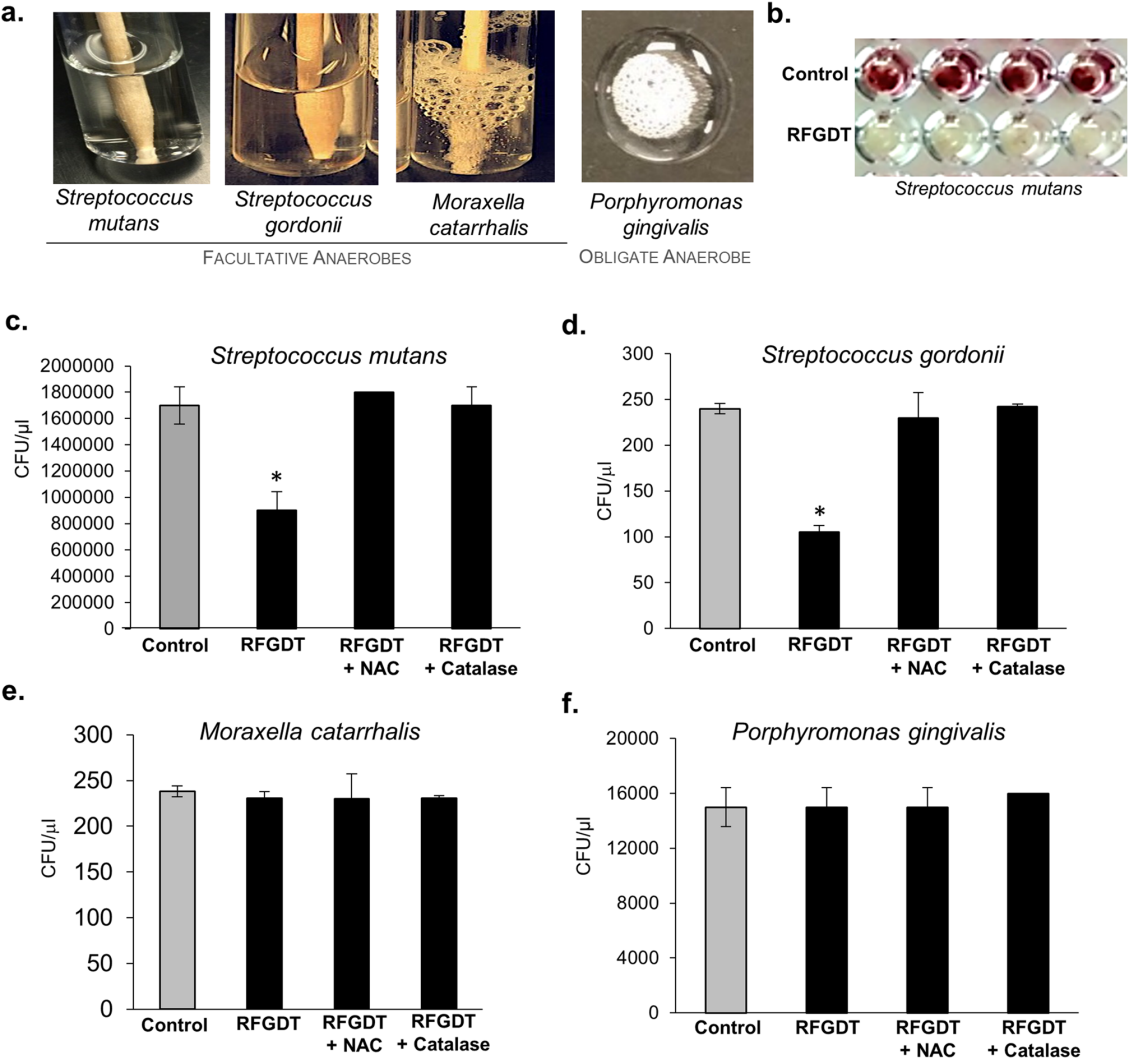
Antimicrobial effects of RFGDT. (**a**) Four bacterial species were subjected to hydrogen peroxide, and effervescence was assessed; (**b**) bacterial viability test with TTC assay was performed with *S. mutans* following RFGDT. Colony-forming assays were performed with four bacterial species following RFGDT namely (**c**) *Streptococcus mutants,* (**d**) *Streptococcus gordonni, (e*) *Moraxella catarrhalis* (**f**) *Porphyromonas gingivalis*. In some cases, incubation with N-Acetyl cysteine or catalase was performed, followed by RFGDT. n = 3, *p < 0.05. RFGDT: Radiofrequency glow discharge treatment; NAC: N Acetylcysteine; CFUs: Colony-forming units.

To examine the disinfection potential of RFGDT, we first examined the endogenous antioxidant by incubating individual microbe with hydrogen peroxide. We noted that *Streptococcus gordonni* and *Streptococcus mutants,* which are catalase-negative, failed to generate effervescence (Fig. 2a). In contrast, *Moraxella catarrhalis* and *Porphyromonas gingivalis,* which are catalase-positive, generated profuse effervescence indicating the active ROS neutralization capabilities. Glass coverslips subjected to RFGDT were seeded with *S. mutant* and demonstrated reduced viability with the live-dead assay (Fig. 2b). Consistent with this observation, colony-forming units (CFUs) of *S. mutants* (Fig. 2c) and *S. gordonni* (Fig. 2d) were significantly reduced following RFGDT. The reduction in microbial survival could be prevented by pre-incubation with ROS scavengers N-acetyl cysteine (NAC) and catalase. Consistent with these observations, no differences in CFUs were observed with *M. catarrhalis* (Fig. 2e), and *P. gingivalis* (Fig. 2f) seeded on glass coverslips following RFGDT. These results demonstrate RFGDT-induced redox reduces viability and proliferation of susceptible (catalase-negative) microbes, while anaerobic, catalase-positive species were resistant.

### RFGDT improves cell adhesion and expansion

**Figure 3:**
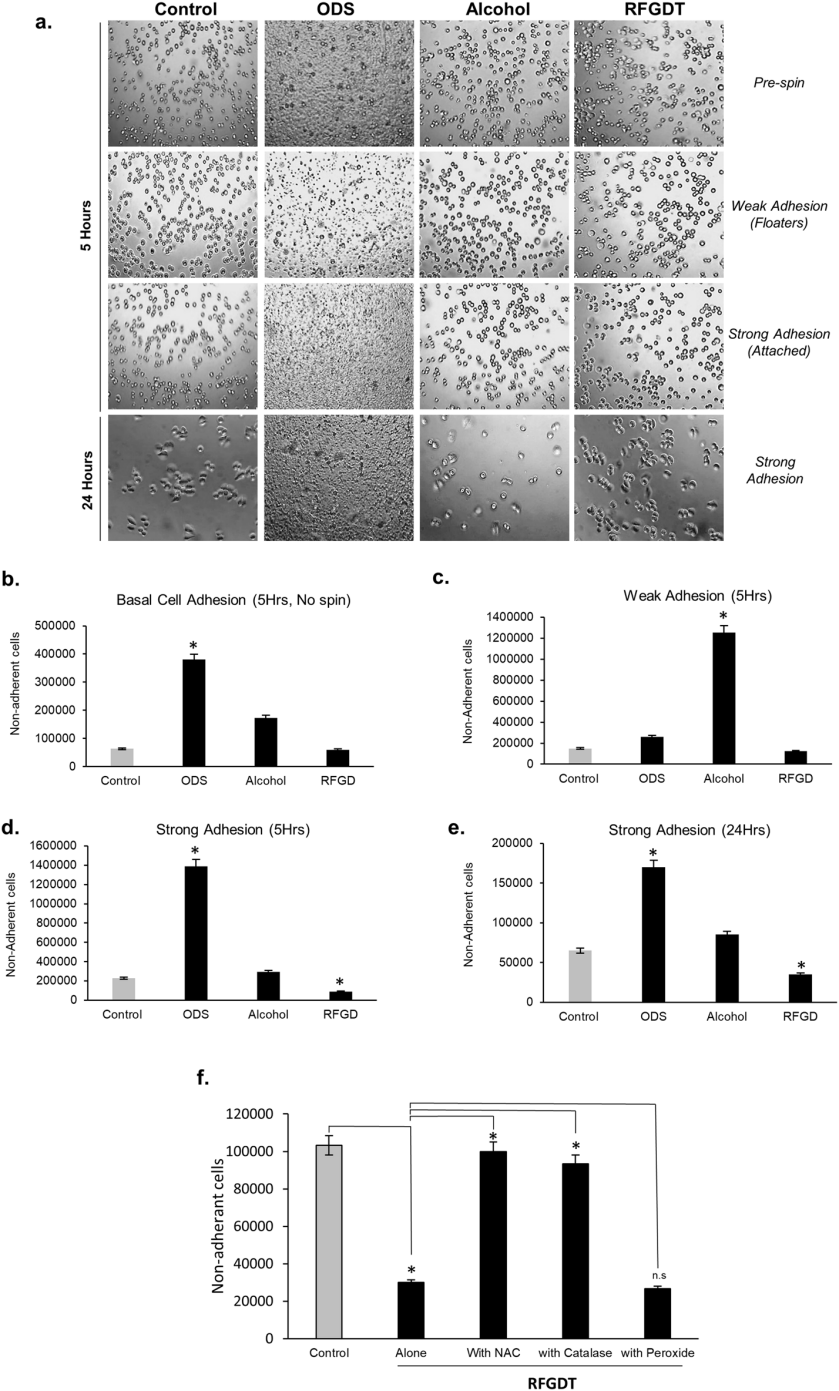
Cell adhesion following RFGDT. (**a**) MDPC-23 cells were seeded on these surfaces and assessed with microscopy; (**b**) Cell adherant to various treatmed biomaterial surfaces were assessed by cell counting; (**c**) Cell-seeded surfaces were subjected to low (2400 rpm) centrifugal forces to determine weak cell adhesions at 5 h; (**d**) Cell-seeded surfaces were subjected to high (4000 rpm) centrifugal forces to determine strong cell adhesions at 5 h and (**e**) 24 h. (**f**) Strong cell adhesion was assessed with glass surfaces pretreated with NAC, Catalase, or hydrogen peroxide before RFGDT. All studies, n = 3, *p < 0.05. RFGD: Radiofrequency glow discharge; ODS: n-Octadecyl Trichlorosilane; RFUs: Relative fluorescence units; NAC: N Acetylcysteine.

Cell adhesion to the substrate is crucial for attachment and subsequent proliferation. Several biomaterials, such as dental cements and composites, include glass or metals that directly contact dental pulp cells. Therefore, we next examined the effects of RFGDT on a mammalian pred-odontoblast cell line, MDPC-23. Glass coverslips were subjected to detergent, alcohol, silane (ODS), or RFGDT prior to seeding. We examined both weak and strong cell adhesions by assessing basal and centrifugal force-induced adherent and floating cell populations at 5 and 24 h after cell seeding. We observed a maximal number of adherent cells on RFGD treatment while most cells on ODS and alcohol-treated surfaces were non-adherent (Fig. 3a, b). Time course analysis after 5 and 24 h with the adhesion strength assays further validated this observation with silane and alcohol-treated surfaces, demonstrating most non-adherent cells in contrast to RFGDT that offered an optimal biomaterial interface for predominantly strong cell adhesion (Fig. 3c–e). The pronounced increase in non-adherent cells in the alcohol-treated group at 5 h could be attributed to a persistent residual agent reducing cell viability. Finally, incubation with either *N*-acetyl cysteine or catalase was noted to specifically reduce RFGDT-improved cell attachment (Fig. 3f). These results suggest that the improved cell adhesion following RFGDT is mediated via redox-mediated signaling.

### Improved cell adhesion is mediated via RFGDT-generated redox-mediated RPSA expression

**Figure 4:**
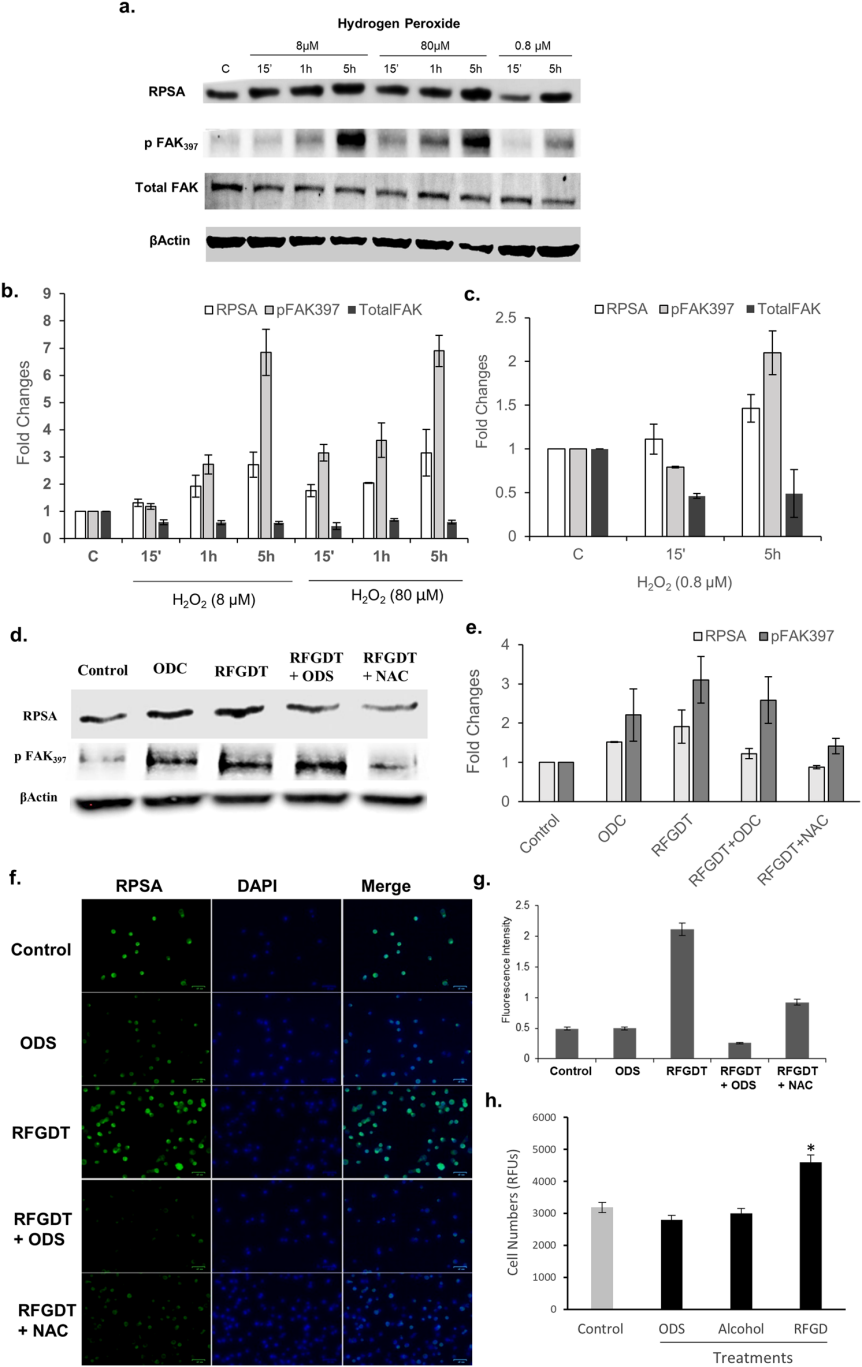
Redox generated by RFGDT induces RPSA expression. (**a**) Western blots for RPSA and activated FAK expression was assessed following hydrogen peroxide treatments over time; (**b**) and (**c**) band quantitation of the western blots is shown; (**d**) Similar analysis was performed following RFGDT on glass surfaces. Some samples were pretreated with ODS or NAC before RFGDT and cell seeding; (**e**) band quantitation of the western blots is shown; (**f**) Immunofluorescence for RPSA expression was performed on these glass surfaces following pretreatments or RFGDT; (**g**) quantification in five high power fields as means with standard deviations; (**h**) Glass surfaces were subjected to silane, alcohol, or RFGDT and seeded with MDPC-23 cells. Cell numbers were assessed with AlamarBlue assay after 24 h. n = 3, *p < 0.05. RFGDT: Radiofrequency glow discharge treatment; ODS: n-Octadecyl Trichlorosilanel; NAC: N Acetylcysteine; DAPI: 4′,6-diamidino-2-phenylindole.

Cell adhesion involves concerted actions of multiple cell membrane and cytoplasmic molecules capable of dynamic assembly of an attachment complex^31–33^. Among these various mediators, laminin receptors on the cell surface have been shown to play a critical role in mediating matrix interactions^37^. Elucidation of these adhesive complexes has also outlined several upstream signaling mediators that outline the role of a laminin receptor, Ribosomal Protein SA (RPSA), induced by redox signaling^34,35^. Hence, to examine the effects of redox signaling on cell adhesion, MDPC-23 cells were treated with various concentrations of hydrogen peroxide that demonstrated increased RPSA and phospho-FAK_397_ expression (Fig. 4a–c). Next, we examined if the RFGDT-improved cell adhesion could be mediated via a similar mechanism. We observed a robust increase in phospho-FAK_397_ and RPSA expression following RFGDT by immunoblotting (Fig. 4d, e) and immunofluorescence (Fig. 4f, g). These responses were abrogated by pretreatment of the biomaterial surface with silane or by adding NAC, indicating these are redox-mediated. Finally, we inquired if the improved cell attachment via the enhanced laminin receptor following RFGDT would affect cell survival and proliferation. We observed a significant improvement (n = 3, *p* < 0.05) in RGFDT group compared to all other groups (Fig. 4h). These results suggest improved cell adhesion following RFGDT could be mediated via induced RPSA expression.

## Discussion

Biomaterial interfaces represent a major frontier for improving functional integration and determining clinical therapeutic outcomes^36^. Significant progress in biomaterial composition and design has evolved from simple biocompatible, inert interfaces to existing bioactive surfaces. More recent biomaterial systems focus on smart systems capable of sense-and-respond to biological scenarios with directed responses. A major functional characteristic of these biomaterial interfaces is to promote desirable biological responses while actively dissuading detrimental ones selectively^3^. Some of the latter responses are directed at reducing microbial (biofilm) burden to prevent protracted inflammation or host immune responses^37,38^. This facilitates routine tissue healing responses and may enable regenerative clinical outcomes.

**Figure 5:**
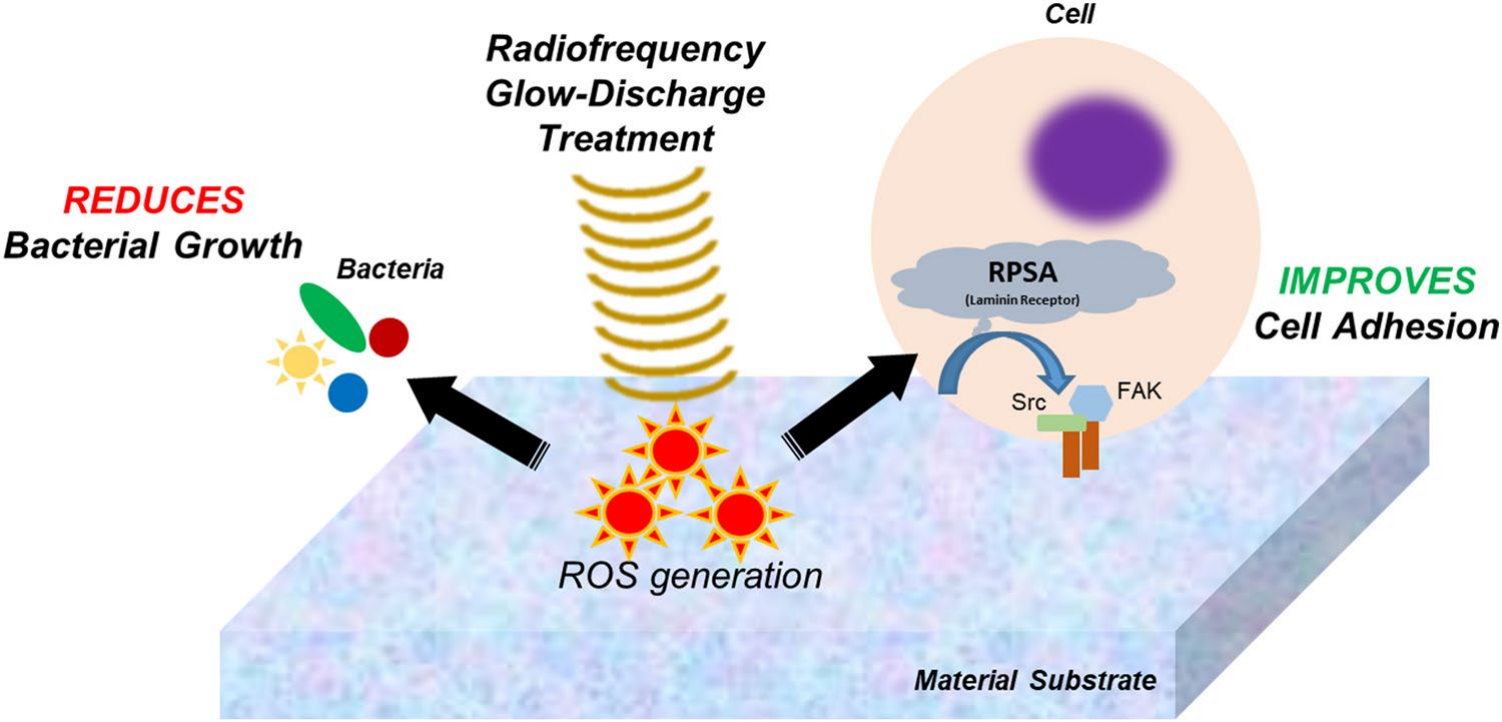
Outline of RFGDT responses. The image shows the dual actions of the redox generated by RFGDT with an antimicrobial effect on oral bacteria while inducing the laminin adhesion receptor, RPSA facilitating improved cell attachment and expansion of odontoblasts.

This study utilized common oral microbes and cells from the dental pulp to examine the effects of RFGDT on biomaterials used routinely in clinical dentistry. Pulpal inflammation due to caries results in irreversible necrosis, necessitating Endodontic intervention and coronal restorations. Various pulp capping agents such as calcium hydroxide, mineral trioxide aggregate (MTA), Emdogain, or glass ionomer cement provide cues to promote tissue healing (osteodentin). However, they do not directly address residual infection that leads to protracted inflammation. Direct plasma surface modification strategies generate antibacterial properties via modulating surface topography or chemistry^39^. Besides serving as an alternate disinfection technique, its effects on improving biocompatible interfaces have been well documented. This prominent dual-action of RFGDT noted in this study, namely antimicrobial disinfection and promoting cell adhesion and expansion, is particularly attractive for clinical use (Fig. 5)^40,41^. The antimicrobial actions of RFGDT predominantly targeting the catalase-negative microbes appear to be directly relevant to healthy clinical scenarios where aerobic bacteria predominate. The reduced efficacy on catalase-negative microbes was evident in this study could be clinically addressed with additional rinses or medications prior to material placement.

This study noted that the generation of redox species from biomaterial surfaces was based on their chemical composition. While both glass and platinum could induce redox, the polymeric (PMMA) substrate demonstrated minimal induction. This appears to reflect the ionization potential of the predominant elemental (Glass: Silica 786 kJ/mol and Platinum 867 kJ/mol) constituents that are representative of dental biomaterials such as reinforced cement and metal implants. Glass or metal reinforced cements and composites are routinely used as dental restorative materials. A prior study noted that Dicor glass-restorations luted with resin composites improved clinical survival than other biomaterials^42^. However, unlike glass and these two biomaterials that intimately interact with cells, the polymeric material (PMMA: 6857 kJ/mol) is predominantly used to fabricate external prosthetic devices where the disinfection non-adhesive characteristics are of most significance. Future investigations could investgate the cellular responses to PMMA including funcationalization approaches to improve cellular interface. Nonetheless, direct composite materials such as pulp dressings would appear unsuitable for the RFGDT technique.

We observed an optimal treatment time of three minutes for disinfection and improved cell adhesion with RFGDT for glass surfaces under vacuum. Besides the biomaterial composition and treatment time, other variables determining these interfacial responses include gaseous composition (such as helium or argon) within the chamber, energy density, and the presence of other trace agents^43,44^. Attention to these parameters can enable the development of future biomaterial-specific RFGDT protocols. It is also important to emphasize that the RFGDT was performed on inanimate biomaterial surfaces, before cell seeding, without direct exposure to microbes or cells. Direct treatments with non-thermal plasma on mammalian cells result in DNA damage and loss of viability^45^. This essentially implies that direct clinical RFGDT on human tissues for disinfection or improving cellular responses is an untenable strategy. Nonetheless, surface treatments of biomaterial devices could prime them for optimal tissue interactions such as osseointegration of metallic implants, glass ionomer, or metal-reinforced composites as dental filling materials. The latter application was a primary motivation in our choice of the pre-odontoblast cell line, MDPC-23. Interestingly, we have observed differential redox sensitivity among cells of discrete lineage in other studies^46^. Future studies could examine the selectivity of RFGDT on complex multipartite biomaterial systems with specific cell types such as osteoblasts (osseointegration), keratinocytes (wound healing), fibroblasts (matrix production), or endothelial cells (angiogenesis) for directed clinical outcomes. This study observed RFGDT-generated ROS detected using chemiluminescence following oxidation of luminol^31^. The amounts and discrete ROS have critical roles in cell survival, migration, and apoptosis^47,48^. Redox is a physiological regulator of intracellular signaling cascades, especially tyrosine kinase-mediated growth factors, has gained recent attention due to its ability to reversibly oxidize redox-sensitive targets^49^. These reversible redox modifications of cysteine-residues in proteins, also termed thiol switches, are regulated by oxidoreductase and thiol-isomerases^50^. Their primary function involves neutralizing excessive redox in the biological system that can lead to deleterious damage. Other targets of redox-generated signaling include a broad array of cytoskeletal components, signaling intermediates, and transcriptional regulators.

A significant finding in this study is that RFGDT induced redox promotes expression of the laminin receptor, Ribosomal Protein SA (RPSA), that contributes to improved cell adhesion and survival. Laminins are high molecular weight extracellular glycoproteins that constitute the major non-collagenous component of basal lamina in cells^51,52^. Their multidomain proteins are encoded by eleven human genes characterized by heterodimeric α, β and γ chains that constitute 16 different isoforms. Their key contributions to cellular functions such as adhesion, migration, and differentiation are well described. There have been recent insights into the roles of specific Laminins to selectively drive cellular enrichment and lineage specification that has potent implications for tissue healing and regeneration^53^. Besides the prototypical members, there are many proteins with laminin-like domains that include RPSA, LamR, 37LRP, and LBP/p40, among others^54^. RPSA is a ribosomal protein with several pathophysiological functions in protein translation, cell adhesion, tumor cell invasion, viral cell entry, and small molecule sensing^55,56^. The amino acid sequence is evolutionarily conserved with over 98%c homology in all mammals and structural similarities with the prokaryotic gene, RPS2^54^. It would be interesting to examine the functional significance of RFGDT induced RPS2 in the microbial species in future studies.

RPSA has been noted to be involved in cap-dependent eukaryotic translation, especially in transformed and viral infected cells. However, upstream induction of RPSA signaling remains to be fully elucidated. A few prior reports have noted that RPSA is induced by redox-generated signaling, suggesting it is a redox-inducible target gene^34,35^. Consistent with these reports, this study outlines the ability of RFGDT induced redox to increase RPSA expression. The increased expression of this cell adhesion receptor would contribute to improved attachment and survival of the MDPC-23 cells. Membrane clustering of these laminin receptors has been noted to induce tyrosine 397 phosphorylation of FAK, as was also noted in this study. Both FAK and Src activation at these membrane adhesion complexes further drive cellular functions such as cell spreading, migration, proliferation, and prevention of apoptosis. Thus, the use of RFGDT appears to evoke discrete redox-generated biological responses that can be harnessed for both disinfection and cellular functions such as cell attachment, expansion, and differentiation to aid tissue regeneration.

In conclusion, this study demonstrates the ability of a non-invasive, directed energy tool, RFGDT, to be capable of concurrent antimicrobial disinfection and improved directed cellular responses via redox-signaling. This showcases RFGDT as a simple, cost-effective, and convenient bench-top tool for clinical application to improve tissue healing and regenerative outcomes.

## Materials and methods

### Cell culture

Mouse dental pulp cells (MDPC-23, kind gift from Drs. Jacques Nor and Tatiana Botero, Universithy at Michigan) were maintained in DMEM (Glutamax, high-glucose) supplemented with 10% Fetal Bovine Serum (certified grade) and 100 units mL^−1^ Penicillin and 100 µg mL^−1^ Streptomycin (all from Thermo-Scientific, USA). Cells were grown at 37 °C in a humidified chamber with 5% CO_2_.

### Sample preparation and sterilization

Cover glass (18 mm, 0.12–0.17 mm thickness, Matsunami micro cover glass) were first cleaned with 10% detergent (Sparkleen, Fisher Scientific, USA) and sonicated (Aristocrat Ultrasonic, Healthco, USA) for 10 min, followed by three washes in distilled water and allowed to dry vertically in a holder. Following this, some covers slips were soaked in 95% Ethanol (Sigma-Aldrich, USA) for 10 min, while another set was soaked in 2% *n*-octadecyl trichlorosilane (ODS) for 10 min. Finally, another set of coverslips was subjected to RFGDT, as described below. Except for the RFGDT, all samples were sterilized under UV light or 15 min before cell seeding.

### Radiofrequency glow discharge treatments (RFGDT)

A partial air-vacuum RFGD device (PDC-32G, Harrick Scientific, USA) was used as per the manufacturer's instructions. The biomaterials (glass coverslips, platinum sheets, or PMMA discs) were placed within the chamber and treated for various times as indicated in individual studies. Biomaterial specimens were used immediately for biochemical, microbiological, or cell adhesion/survival studies.

### AlamarBlue assay

MDPC-23 cells were seeded at a cell density of 1 × 10^5^ cells mL^−1^ on the treated glass surfaces described above placed in a 12 well dish. Cell viability and proliferation were assessed with the AlamarBlue assay as per the manufacturer's instructions. Briefly, following 24 h of cell incubation, 10% (v/v) of AlamarBlue reagent (Thermo Fisher Scientific, USA) was added to complete media and incubated for 2 h. Replicates of conditioned media from each well were transferred to black wells, and fluorescence was assessed using a plate reader (Victor3, Perkin Elmer, USA).

### Cell adhesion assay

MDPC-23 cells were seeded at a cell density of 1 × 10^5^ cells mL^−1^ on the treated glass surfaces described above placed in a 12 well dish. Cell adhesion was assessed after 5 h of incubation as described previously. As equivalent cells were seeded on all substrate conditions and no proliferation is expected within 5 h, the non-adherent, floating cells (higher, more reliable global numbers) in each condition were counted rather than imaging multiple high-power fields (lower, less reliable local numbers) per well. Media from each well was collected, stained with Trypan Blue (0.4% w/v, Sigma-Aldrich, USA), and assessed for floating (non-seeded) cells. After replacing the media, plates were subjected to centrifugation (Eppendorf, USA) at 2400 rpm (weak adhesions) and 4000 rpm (strong adhesions) for 6 min, and the media was collected to assess cell numbers. Images were also captured of these wells before and after spinning to document the number and morphology of the adherent cell populations. In some studies, Catalase (10 uM), *N*-acetyl cysteine (1 mM), or hydrogen peroxide (all from Sigma-Aldrich, USA) were included in the media prior to cell seeding of the glass surfaces to assess the role of RFGDT-induced ROS in mediating cell adhesion.

### ROS detection assay

To assess RFGDT-generated ROS, a chemiluminescence assay with Luminol (detects both extra- and intracellular ROS) was utilized as described previously^33^. Briefly, a solution was prepared by mixing 5 mg of luminol powder with 100 mL of 1 M Sodium hydroxide (both Sigma-Aldrich, USA) and mixed gently. The freshly prepared luminol solution was added to the cells in a 12 wells plate at a ratio of 1 mL/well (v/v) immediately after RFGDT treatments at different doses, and chemiluminescence was detected at 0.5, 1, 3, and 5 min using a multiwell detector (Victor3, Perkin Elmer, USA).

### Western blotting

Glass coverslips were RFGDT treated and seeded with MDPC23 cells. After 5 h of incubation in CO_2_ incubator, the attached cells on the coverslip were collected from different groups, and collected cells were re-suspended in RIPA buffer (Sigma-Aldrich, USA) with Mini Protease Inhibitor (Thermo Scientific, USA). Cells were disturbed with two cycles of sonication (QSonica, USA) for 5 s each, and lysates were centrifuged at 14,000 rpm at 4 °C for 20 min. The total protein in the lysates was quantified with the Bradford assay kit (BCA Protein Assay, Thermo Scientific Inc). Protein lysates were separated in precast mini-protein TGX stain-free gels and transferred to PVDF membranes (both Bio-Rad, USA). Blots were initially blocked with 1% BSA for 1 h and further incubated with primary antibodies for Ribosomal Protein SA (RPSA, Abcam, USA) or phospho-FAK (Cell signaling, USA) at 4 °C overnight. Following washes, blots were incubated for normalization with appropriate species-specific HRP conjugated secondary antibodies (Cell signaling, USA) or HRP-conjugated β-actin (Cell signaling, USA). Finally, the blots were developed by using chemiluminescent substrates (Thermo Scientific, USA), and images were scanned digitally using ChemiDoc MP Imaging System (Bio-Rad Laboratories, USA). Western blots protein band intensities were quantified by using ImageJ software. Multiple blot exposures are provided in supporting figures.

### Immunofluorescence staining

In all groups, immunofluorescent experiments were carried out using primary laminin antibody (RPSA) (Cat # ab133645, Abcam, USA) and Alexa fluor 488 secondary antibody (Cat # 4412S, Cell signaling, USA), image were captured by using ZOE Fluorescent Cell Imager (Bio-rad, USA), where nucleus was counterstained stained with DAPI. Digital images were captured, and following thresholding, fluorescence intensity over the entire field was quantified using NIH ImageJ software as described previsouly^57,58^.

### Microbial viability assay

Facultative anaerobes such as *Streptococcus mutants* (*S. mutants*), *Streptococcus gordonii* DL1 (*S. Gordonni*), *Moraxella catarrhalis* (*M. catarrhalis*) were grown in brain heart infusion medium (BD Bioscience, USA) at 37 ℃ and Obligate anaerobe *Porphyromonas gingivalis* (*P. gingivalis*) was grown anaerobically in trypticase soy broth medium (BD Bioscience, USA) supplemented with 10% sheep blood, hemin (5 μg mL^−1^) and menadione (1 μg mL^−1^) and incubated at 37 °C in an atmosphere of N_2_/H_2_/CO_2_ (90:5:5). A single colony of each bacterium was added to 5% or 15% of H_2_O_2_ and qualitatively analyzed for the presence of antioxidant catalase. For the survival assay, a 10% 2,3,5-Triphenyl-tetrazolium chloride solution (TTC, Sigma Aldrich, USA) was added to cultures wells and incubated for 6–12 h or until red color change was apparent. Color intensity was quantified by using a microplate reader (SpectraMax i3x, Molecular device, USA) at 480 nm.

### Microbial survival assay

For the CFU assays, coverslips were inoculated with 250 uL respective bacterial culture and incubated in species-specific growth conditions for 24 h (*S. gordonni*, *M. catarrhalis*, *S. mutants*) or 5 days (*P. gingivalis*). Aliquots of bacterial culture from glass coverslips were serially diluted by tenfold dilutions and plated via the drop plate technique.

### Statistical analyses

Data were analyzed using Excel (Microsoft, USA), and statistical significance was assessed using either a one-way ANOVA or *t* tests, where *p* < 0.05 was considered statistically significant. All studies were repeated at least twice, with each assay performed in minimum duplicates.

## Supplementary Information

Supplementary Information.

## Data Availability

All data generated or analyzed during this study are included in this published article and its supplementary information files. However, any further information or material is available from the authors upon reasonable request.
