# Supplementary material for: Redox signaling induces laminin receptor ribosomal protein-SA expression to improve cell adhesion following radiofrequency glow discharge treatments
Source: Sci Rep. 2022 May 11;12:7742. doi: 10.1038/s41598-022-11766-9 (PMC9095671; doi:10.1038/s41598-022-11766-9)

SUPPORTING FIGURE LEGENDS

Supporting Figure 1a. The western blot images in Fig 4A are correlated with entire blot images of approrpaite exposure with individual lanes clearly dileanated.

Supporting Figure 1b. Multiple exposures for each western blot is presented for comparison.

Supporting Figure 2a. The western blot images in Fig 4D Multiple exposures for each western blot is presented for comparison. Note, the first and last lane represnting non-relevant controls are omitted, and only the five central lanes are presented.

Supporting Figure 2b. Multiple exposures for each western blot is presented for comparison.

Supporting Figure 1a

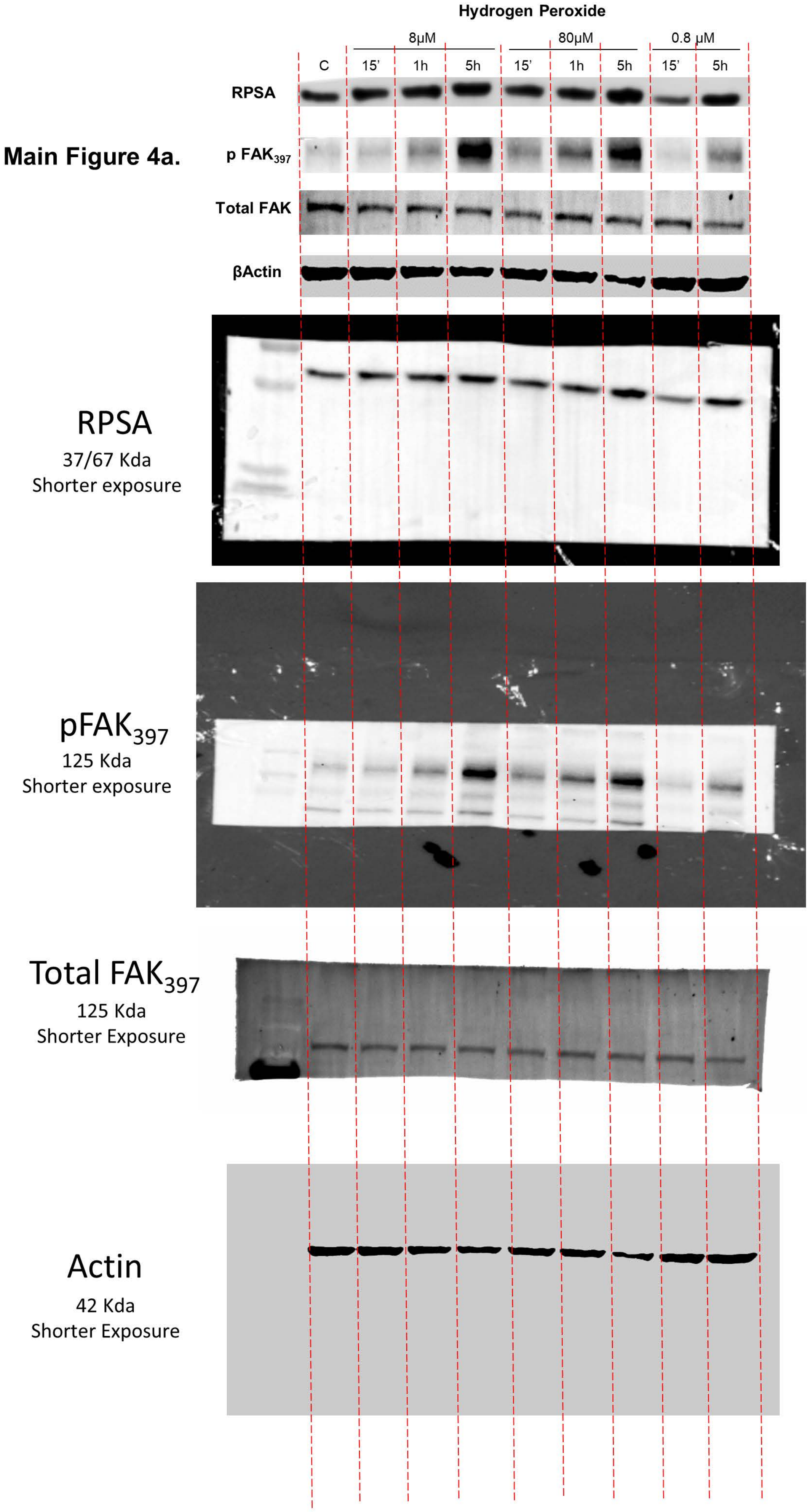

Supporting Figure 1b

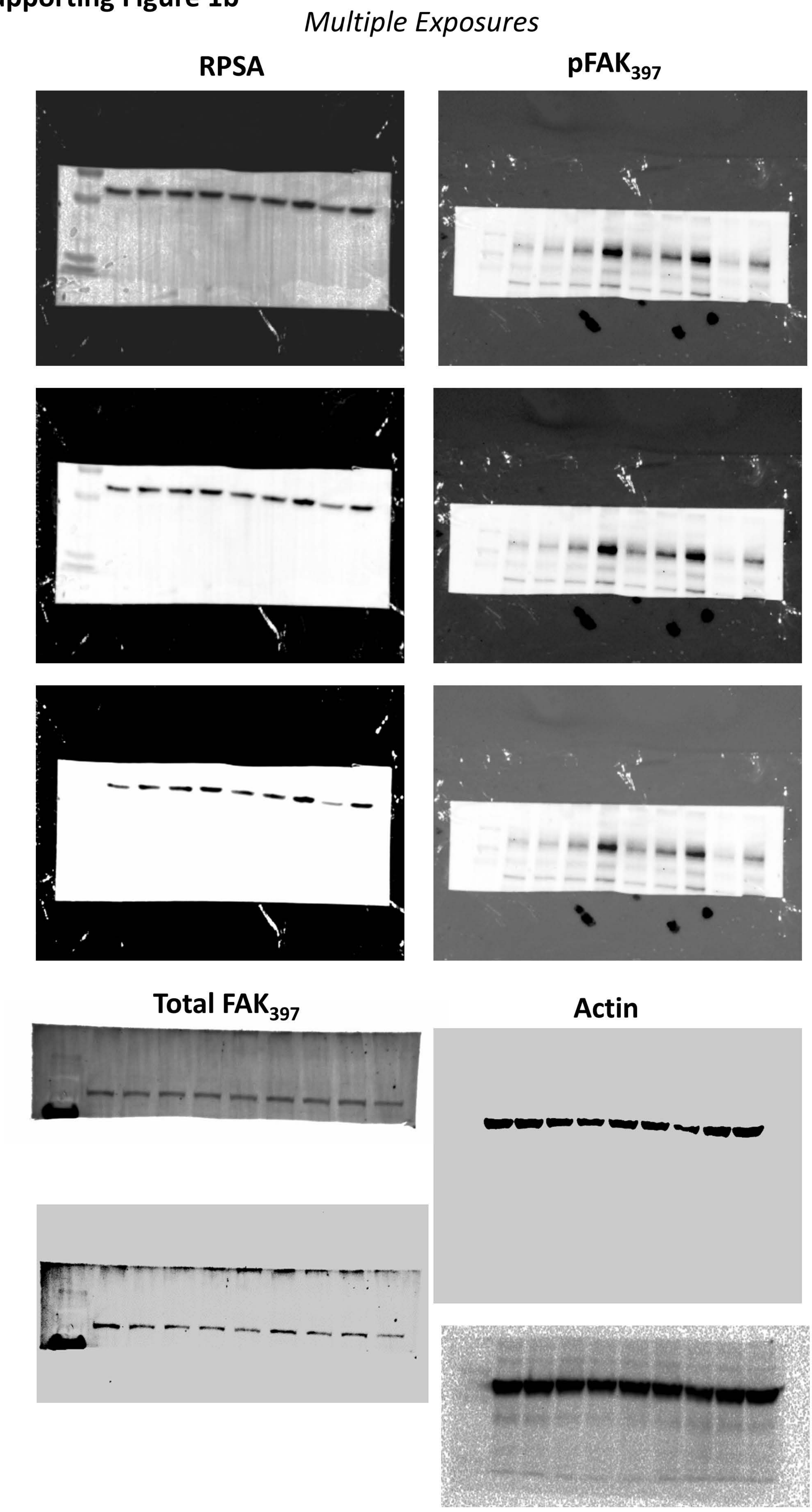

Supporting Figure 2a

Main Figure 4d.

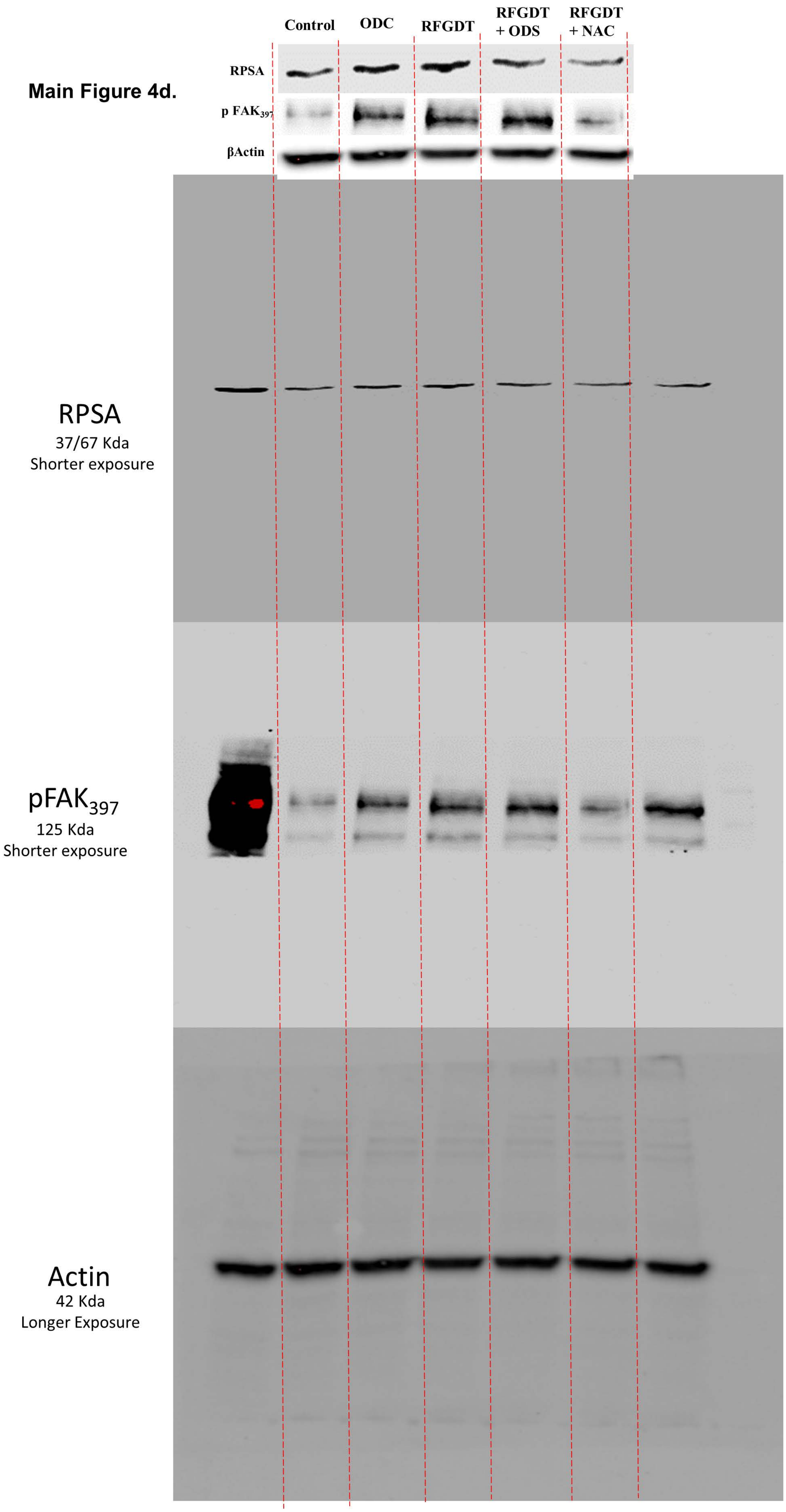

Supporting Figure 2b

Multiple Exposures

RPSA

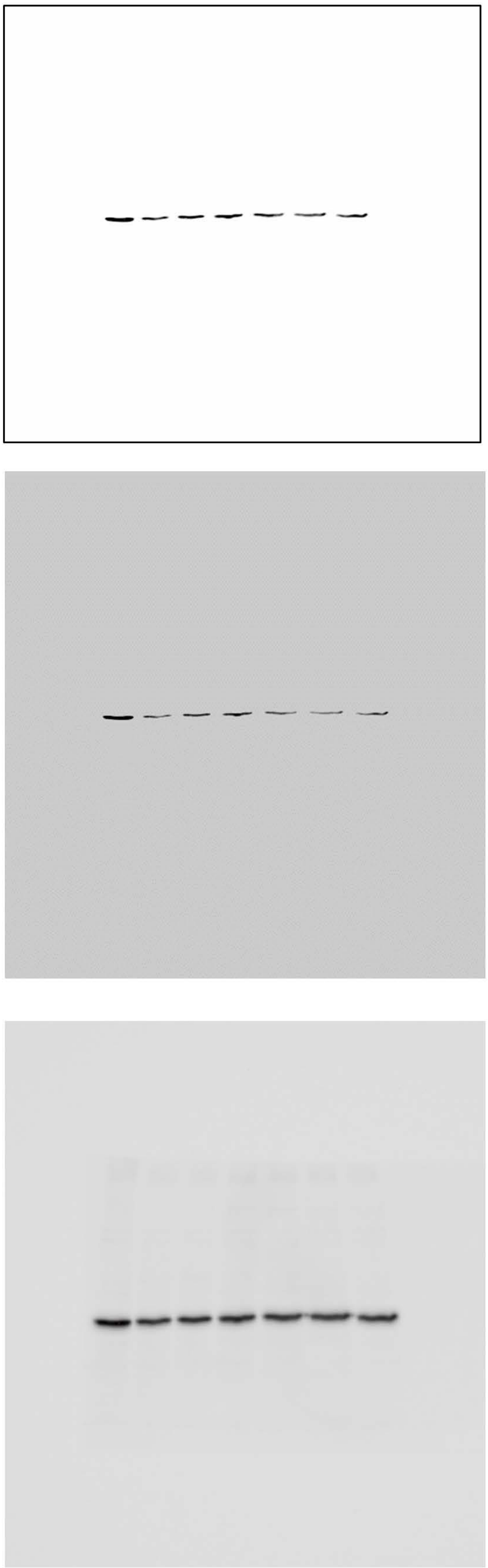

pFAK$_{397}$

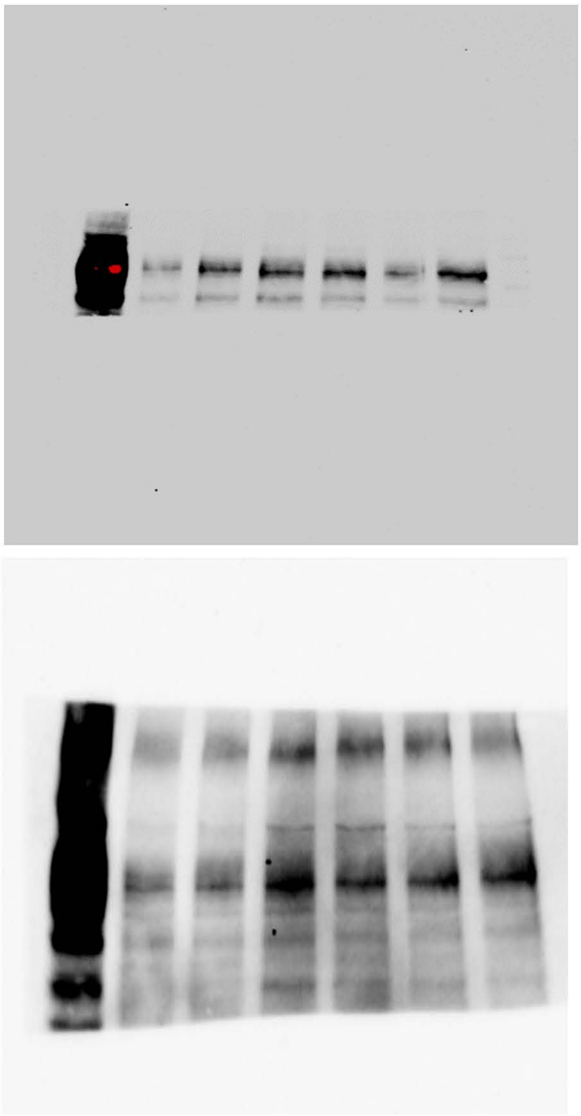

Actin

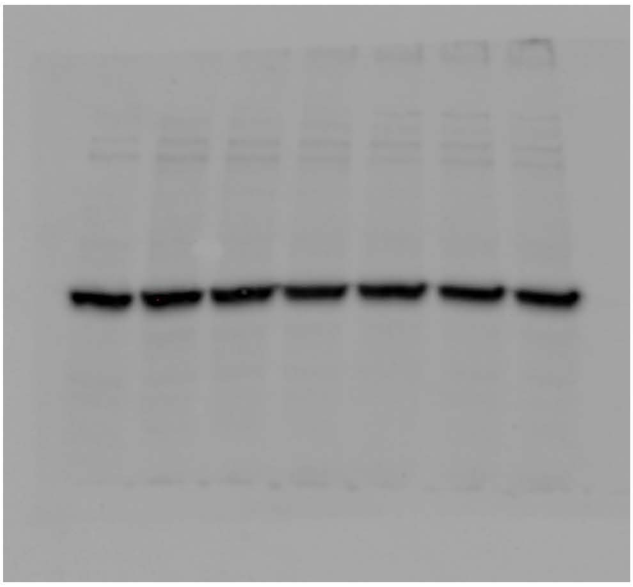

Supplement: Supplementary file 1 — Supplementary Information. [file 41598_2022_11766_MOESM1_ESM.pdf]
